# Canagliflozin Prevents Lipid Accumulation, Mitochondrial Dysfunction, and Gut Microbiota Dysbiosis in Mice With Diabetic Cardiovascular Disease

**DOI:** 10.3389/fphar.2022.839640

**Published:** 2022-02-23

**Authors:** Xueliang Wang, Zhe Wang, Di Liu, Hao Jiang, Chao Cai, Guoyun Li, Guangli Yu

**Affiliations:** ^1^ Key Laboratory of Marine Drugs of Ministry of Education, Shandong Provincial Key Laboratory of Glycoscience and Glycotechnology, School of Medicine and Pharmacy, Ocean University of China, Qingdao, China; ^2^ Precision Medicine Institute, The First Affiliated Hospital of Sun Yat-Sen University, Sun Yat-Sen University, Guangzhou, China; ^3^ Laboratory for Marine Drugs and Bioproducts, Pilot National Laboratory for Marine Science and Technology, Qingdao, China

**Keywords:** canagliflozin, mitochondrion, hypolipidemic, colonic microbiota, myocardial protection, diabetic cardiovascular disease

## Abstract

Type 2 diabetes mellitus (T2DM) is associated with cardiovascular disease (CVD) and sodium glucose cotransporter 2 inhibitors, as oral medications for T2DM treatment have shown the potential to improve vascular dysfunction. The aim of this study was to evaluate the ability of canagliflozin (Cana) to relieve CVD in T2DM mice and its possible action mechanism. Mice with diabetic CVD was conducted by a high-fat diet for 24 weeks, followed by oral gavaging with metformin (200 mg/kg/day) or Cana (50 mg/kg/day) for 6 weeks. The result demonstrated that Cana reduced serum lipid accumulation, and decreased the arteriosclerosis index and atherogenic index of plasma. In addition, Cana treatment reduced the circulating markers of inflammation. More importantly, Cana improved cardiac mitochondrial homeostasis and relieved oxidative stress. Moreover, Cana treatment alleviated the myocardial injury with decreasing levels of serous soluble cluster of differentiation 40 ligand and cardiac troponin I. Thus, cardiovascular abnormality was relieved by suppressing fibrosis and basement membrane thickening, while elevating the cluster of differentiation 31 expression level. Importantly, Cana increased the ratio of gut bacteria *Firmicutes/Bacteroidetes* and the relative abundance of *Alistipes*, *Olsenella*, and *Alloprevotella*, while it decreased the abundance of *Mucispirillum*, *Helicobacter*, and *Proteobacteria* at various taxonomic levels in mice with diabetic CVD. In short, Cana treatment altered the colonic microbiota composition close to the normal level, which was related with blood lipid, inflammation, and oxidative stress, and might play a vital role in CVD. In general, the improvements in the gut microbiota and myocardial mitochondrial homeostasis may represent the mechanism of Cana on CVD treatment.

## Introduction

Individuals with type 2 diabetes mellitus (T2DM) are at high risk for macrovascular complications (such as cerebrovascular and cardiovascular) (DeFronzo et al., 2015). T2DM is associated with cardiovascular disease (CVD) characterized by increases in endothelial dysfunction and large artery stiffness (Figueiredo et al., 2012). Risk factors of CVD include hyperglycemia, dyslipidemia, insulin resistance (IR), obesity, hypertension, nonalcoholic fatty liver disease, and so on (Brindle et al., 2006; Stevens et al., 2016). Although there is a correlation between CVD event rates and magnitude of hyperglycemia (Gerstein et al., 2005; Fox et al., 2015), there remains some nondeterminacy as to whether antidiabetic medications can reduce CVD risk (Group, 2008; Skyler et al., 2009). Dyslipidemia, oxidative stress, and inflammation are vital factors in the development of diabetic CVD (Nishikawa and Araki, 2007; Braunwald, 2008; Tietge, 2014). It was demonstrated that high-fat diet (HFD)-induced elevated cholesterol and low-density lipoprotein cholesterol (LDL-C) resulted in both ischemic heart diseases with or without inflammation (Varbo et al., 2013). Hyperlipidemia is an elevation of lipids in the bloodstream (Jain et al., 2007), which is a cause of atherosclerosis and atherosclerosis-associated conditions (Aronson and Rayfield, 2002). Moreover, mitochondria are the most important sources of reactive oxygen species (ROS), and mitochondrial dysfunction increases electron leak and ROS production (Kayama et al., 2015).

In recent years, sodium glucose cotransporter 2 inhibitors (SGLT2i) were introduced as T2DM treatment (Rosenwasser et al., 2013). In meta-analyses of clinical trials in T2DM, three types of SGLT2i, including canagliflozin (Cana), dapagliflozin (Dapa), and empagliflozin (Empa) could reduce fasting plasma glucose and hemoglobin A1c (HbA1c) (Liakos et al., 2014; Sun et al., 2014; Yang et al., 2014). Moreover, it has been reported that several SGLT2i showed notable beneficial effects on CVD unlike other classes of antidiabetic medications (Dziuba et al., 2014; Lee et al., 2018; Jiang et al., 2021; Tan et al., 2021; Xiang et al., 2021).

In recent years, associations between T2DM and altered gut microbiome composition have been reported (Forslund et al., 2015). Moreover, many research indicate that the gut microbiota has emerged as a vital regulator of metabolic diseases, including in cardiovascular function in T2DM (Boulangé et al., 2016; Vikram et al., 2016; Battson et al., 2017). It has been verified that Dapa induced subtle alterations in intestinal flora in T2DM mice (Lee et al., 2018).

Cana was recently developed as a type of SGLT2i to improve T2DM and related diseases in an insulin-independent manner (Zinman et al., 2016; Steven et al., 2017). It has been reported that only Cana marked activated AMP-activated protein kinase (AMPK), but not Dapa, or Empa, which indicated a potential additional effect of Cana compared with other SGLT2i (Hawley et al., 2016). In addition, Cana decreased serum leptin and interleukin-6 (IL-6), while it increased serum adiponectin and tumor necrosis factor alpha (TNF-alpha) in T2DM patients, which favorably impacted insulin sensitivity and CVD risk (Garvey et al., 2018). Recently, several clinical studies indicated that cardiovascular events were lower after Cana treatment in T2DM patients (Neal et al., 2017; Mahaffey et al., 2018; Perkovic et al., 2019; Tian et al., 2021). In addition, Cana significantly altered cecal microbiota composition in renal failure mice and decreased the levels of microbiota-derived uremic toxins in chronic kidney disease (Mishima et al., 2017).

Here, we evaluated the effects of Cana on attenuating CVD in mice with T2DM induced by HFD (Supplementary Figure S1). Fasting blood glucose (FBG) and HbA1c levels were markedly increased after HFD was fed for 24 weeks compared with the NCD-fed group (Supplementary Figure S2). Meanwhile, oral glucose tolerance test (OGTT) and intraperitoneal insulin tolerance test (IPITT) assays demonstrated that the HFD-fed significantly decreased glucose tolerance (Supplementary Figure S2). These results demonstrated that mice with T2DM was successfully induced. Meanwhile, metformin (Metf), as a first-line clinical drug for T2DM, revealed marked improvement in reduction of CVD events (Fung et al., 2015). Thus, Metf was selected as the positive control. With the above background, the purpose of our study was to assess the effects of Cana on hyperlipemia, systemic inflammation, oxidative stress, IR, myocardial mitochondria, and vascular basement membrane in T2DM mice, and the effects of Cana on the gut microbiota dysbiosis was also characterized.

## Materials and methods

### Chemicals and materials

Metf and Cana were purchased from Aladdin (Aladdin Technology Co., Shanghai, China) and Selleck Chemicals (Selleck Chemicals, Houston, TX, USA) separately. All other chemicals were purchased from Sigma-Aldrich (St. Louis, MO, USA) unless otherwise noted.

### Animals and experimental design

The experimental protocol is shown in Supplementary Figure S1. Briefly, male C57BL/6J mice aged 6 weeks with weights of 20–22 g were obtained from the Charles River (Beijing, China). Mice were allowed to acclimatize in the laboratory environment for 1 week before the beginning of the experiment. All mice had free access to food and water. Then mice were randomly divided into four groups (*n* = 10 per group): normal-chow diet (NCD)-fed group (control group) (D12450B, Research Diets Inc., New Brunswick, NJ, USA), HFD-fed group (model group) (D12492, Research Diets Inc.), HFD plus Metf group (Metf group), and HFD plus Cana group (Cana group). Throughout the establishment of T2DM mice model period of 24 weeks, most individuals with HFD feeding appeared to be significantly hyperglycemic compared with the NCD feeding mice, while a few mice maintained a relatively normal blood glucose level. Thus, we randomly selected six mice with hyperglycemia for the following treatment trial. The sample size of each group was calculated by the G*Power software. In a one-way ANOVA with an effect size of *f* = 0.7, *α* of 0.05, power level of 80%, and group number of 4, it was calculated that a number of six mice per group would be sufficient to reach statistical significance. After that, the Metf and the Cana groups were gavaged with Metf (200 mg/kg dissolve in saline solution containing 0.025% Tween-20 and 0.5% carboxymethyl cellulose) or Cana (50 mg/kg dissolve in saline solution containing 0.025% Tween-20 and 0.5% carboxymethyl cellulose) once a day for six more weeks, while the control group and the model group were given equal amounts of solvents as a control vehicle. The body weight was weighed weekly. At the end of the experiment, mice were fasted for 12 h and were euthanized by pentobarbital sodium (100 mg/kg, ip) (Arjinajarn et al., 2016; Thongnak et al., 2017; Jaikumkao et al., 2018).

### Automatic hematological analysis

Blood samples were collected in 1.5-ml K_3_EDTA-containing tubes (Sarstedt, Nümbrecht, Germany) *via* the eyeball method, then analyzed by an automated veterinary hematology analyzer (Mindray, Shenzhen, China).

### Tissue sampling and histological study

Cardiac tissues were collected, then partial cardiac tissues were fixed in a 4% paraformaldehyde solution or 2.5% glutaraldehyde for 48 h, while frozen tissues were used for Oil Red O staining. Then tissues were dehydrated and embedded, and cut into sections. The tissue sections were analyzed by Oil Red O, H&E, or Masson’s trichrome staining (Goldner, 1938; Fischer et al., 2008; Wang et al., 2016).

### Biochemical assays

FBG was measured after fasting for 12 h. Oral glucose tolerance test (OGTT) and intraperitoneal insulin tolerance test (IPITT) assays were conducted (Fraulob et al., 2010; Fei and Zhao, 2013). The increment of blood glucose after glucose loading was expressed in terms of area under the curve (AUC). Blood was collected and centrifuged at 375 × *g* for 10 min to obtain serum. Hemoglobin A1c (HbA1c) and insulin in serum were determined using HbA1c and insulin ELISA Kits (OM549127 and OM451187; Omnimabs, Alhambra, CA, USA), respectively. Homeostasis model assessment-insulin resistance (HOMA-IR) was calculated as fasting insulin × fasting glucose / 22.5. Serum triglyceride (TG), total cholesterol (TC), high-density lipoprotein cholesterol (HDL-C), and low-density lipoprotein cholesterol (LDL-C) levels were detected by assay kits (F001-1-1, F002-1-1, A112-1-1, and A113-1-1; Jiancheng Bioengineering Institute, Nanjing, China). IL-6, monocyte chemotactic protein 1 (MCP-1), and TNF-alpha levels were determined using ELISA kits (M6000B, 479-JE-010/CF, DY410-05; R&D systems, Minneapolis, MN, USA). Soluble cluster of differentiation 40 ligand (sCD40L) and cardiac troponin I (cTn I) contents were determined using ELISA kit (OM448329 and OM453121; Omnimabs, Alhambra, CA, USA). Cardiac tissues (200 mg) were homogenized in cold phosphate buffer, then centrifuged to collect the supernatant. Glutathione (GSH), superoxide dismutase (SOD), malondialdehyde (MDA), and catalase (CAT) contents in cardiac tissue were determined (A006-2-1, A001-3-2, A003-4-1, and A007-1-1; Nanjing Jiancheng Bioengineering Institute, Nanjing, China). In addition, ROS content in cardiac tissue was detected by fluorescent dye dihydroethidium (DHE) as previously described (Zheng et al., 2018).

### Immunohistochemistry and immunofluorescence assays

Cluster of differentiation 31 (CD31) (AF6191, Affinity Biosciences, Cincinnati, OH, USA) and heme oxygenase 1 (HO-1) (86806S, Cell Signaling Technology, Danvers, MA, USA) in cardiac tissues were determined as previously described (El-Azab et al., 2012). After blocking with goat serum, sections were incubated with primary antibody at 4°C for 12 h and then incubated at room temperature for 1 h with fluorescent secondary antibody. Then the sections were stained with DAPI for 30 min, and images were acquired.

### TdT-mediated fluorescein nucleotide (cyanine 3-dUTP) nick-end labeling detection

TdT-mediated fluorescein nucleotide (cyanine 3-dUTP) nick-end labeling (TUNEL) was conducted using the TUNEL Assay Kit (ab66110, Abcam, Cambridge, UK) to evaluate the apoptosis of cardiac tissues. the incorporated fluorescein-labeled dUTP was analyzed.

### Transmission electron microscope

Transmission electron microscopy (TEM) of cardiac tissues was conducted as previously described (Das et al., 2017). Cardiac tissues were fixed in 2.5% glutaraldehyde for 48 h at room temperature. Tissues were embedded in araldite, then cardiac microvasculature and mitochondria were observed by an electron microscope. The vascular basement membrane thickness of each sample was determined by the average value of six measurements at various position.

### Colonic microbiota high-throughput sequencing

Total bacterial DNA was isolated from colonic contents by the QIAamp DNA Stool Mini Kit (51604, Qiagen, Hilden, Germany). The colonic bacterial 16S rDNA gene was amplified by PCR, then the PCR products were purified by the QIAquick Gel Extraction Kit (28706) and sequenced by Realbio (Realbio Technology Co., Ltd., Shanghai, China). Linear discriminate analysis effect size (LEfSe) was used to characterize the significant differences in the abundance of microbial taxa between different treated groups. Heatmap was analyzed by R Cytoscape. In addition, β-diversity was analyzed by the QIIME software.

### Western blot analysis

Western blot analysis was conducted as in our previous study (Wang et al., 2021). In addition, the membranes were incubated with primary antibodies directed against pAMPK (2,531), AMPKα (2,532), and β-actin (4,970) from Cell Signaling Technology (Danvers, MA, USA). The ImageJ software was used for densitometry analysis.

### Statistical analysis

Data are presented as the mean ± standard error of the mean (SEM). The differences between groups were analyzed by GraphPad Prism software using Student’s *t*-test, one-way, or two-way ANOVA as appropriate (San Diego, CA, USA). Differences were statistically significant when *p* < 0.05. In addition, microbial community β- and α-diversity were calculated using Chao1, Shannon, and Simpson indices, and Bray–Curtis distances were visualized by PCoA. The LEfSe characterized a value of *p* < 0.05 and an LDA score > 2.

## Results

### Effects of Cana on hyperlipemia

**FIGURE 1:**
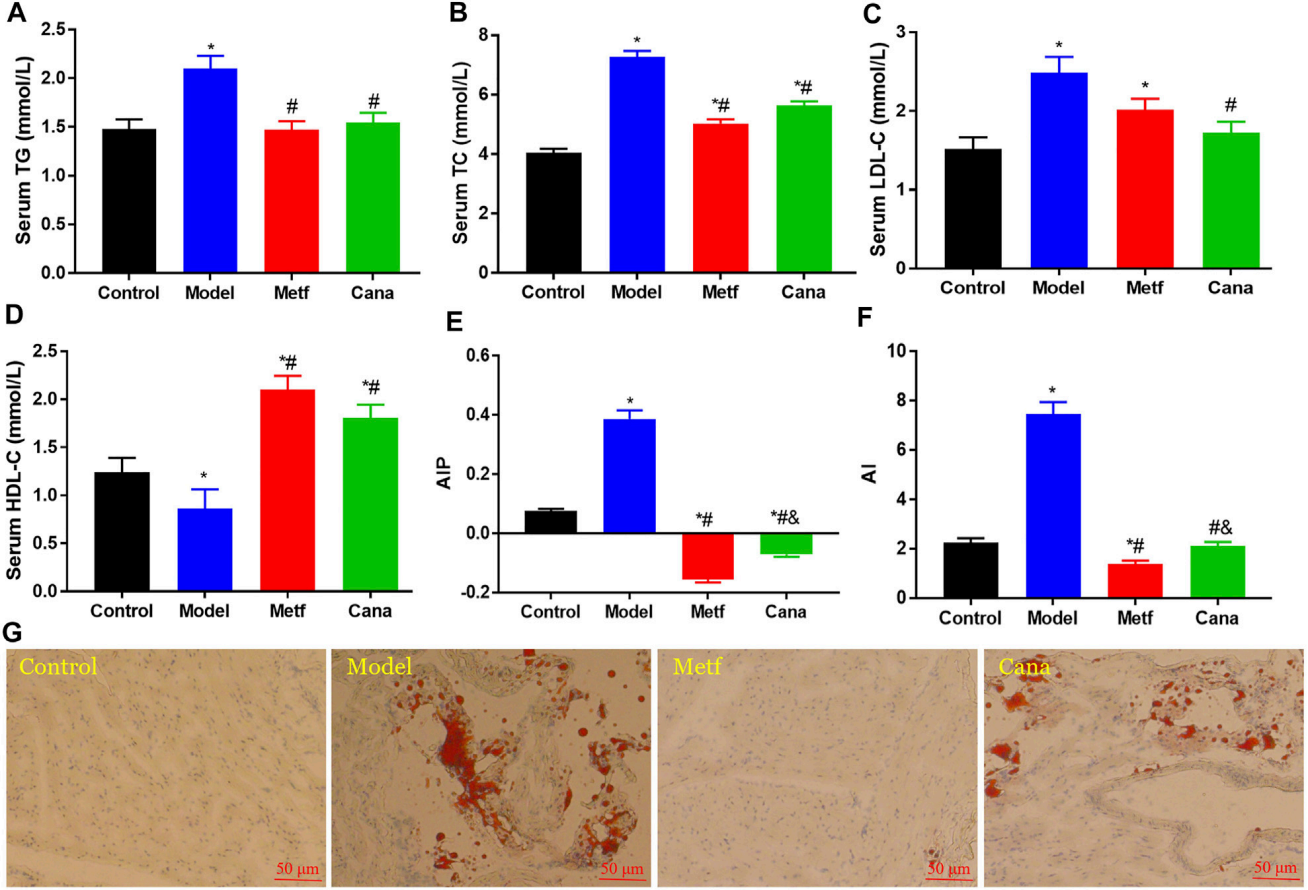
Effects of canagliflozin (Cana) on lipid profile in type 2 diabetes mellitus (T2DM) mice. Triglyceride (TG) **(A)**, total cholesterol (TC) **(B)**, low-density lipoprotein cholesterol (LDL-C) **(C)**, and high-density lipoprotein cholesterol (HDL-C) **(D)** levels in serum. Atherogenic index of plasma (AIP) **(E)**, calculated by lg(TG / HDL-C). Atherogenic index (AI) **(F)**, calculated by (TC − HDL-C) / HDL-C. Cardiac tissues with Oil Red O staining **(G)**. Data are expressed as mean ± SEM. ^&^ *p* < 0.05, the Cana group compared with the Metf group; ^#^ *p* < 0.05, compared with the model group; *p < 0.05, compared with the control group. Six mice in each group were randomly selected, and statistical analyses were performed.

First, we demonstrated that Cana significantly improved blood glucose and insulin homeostasis in mice with T2DM (Supplementary Figure S2). Moreover, Metf treatment remarkedly decreased body weight, body weight gain, and BMI after fed with HFD (Supplementary Figure S3) (*p* < 0.05), while Cana treatment could not. In addition, evidence demonstrates that increased concentrations of cholesterol and TG are causal risk factors for CVD (Nordestgaard and Varbo, 2014). Hyperlipidemia is a vital factor for T2DM-related CVD (Carr and Brunzell, 2004). Thus, managing hyperlipidemia is an effective way to prevent CVD. Our results showed that HFD feeding markedly elevated the levels of TC, TG, and LDL-C in serum (*p* < 0.05) (Figures 1A–C) compared with the control group. However, serous TG, TC, and LDL-C levels in the Cana group were markedly reduced (*p* < 0.05) (Figures 1A–C). In addition, serum HDL-C level increased in the Cana and Metf groups compared with the Model group (Figure 1D) (*p* < 0.05). Atherogenic index of plasma (AIP) and atherogenic index (AI) values increased with the elevated CVD risk, which are also highly sensitive markers of lipoprotein profiles in CVD patients (Dobiásová, 2006). Cana decreased the AIP and AI levels in T2DM mice (Figures 1E, F), which demonstrated that Cana relieved CVD-induced hyperlipidemia in mice with T2DM. In addition, Oil Red O staining indicated that abnormal lipid accumulation was observed in the model group, while treatment with Cana alleviated the lipid accumulation in cardiac tissues (Figure 1G). In addition, we demonstrated the activation of AMPK in cardiac tissues after Cana treatment (Supplementary Figure S4), which may contribute to decrease the lipid levels. Taken together, the above results demonstrated that Cana can perform an antiCVD effect via suppressing lipid accumulation in serum and cardiac tissue in T2DM mice.

### Effects of Cana on oxidative stress and systematic inflammation in type 2 diabetes mellitus mice

**FIGURE 2:**
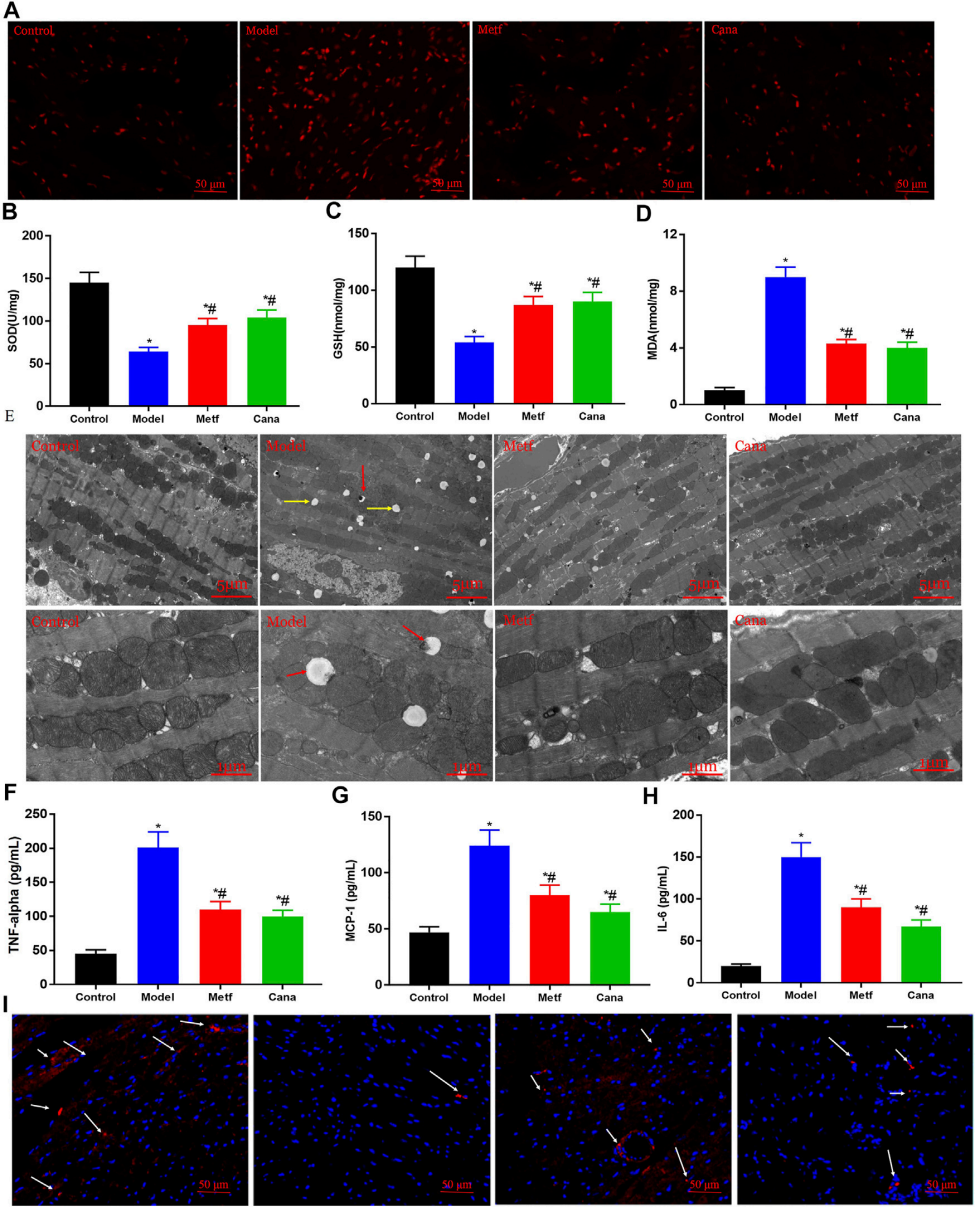
Effects of Cana on alleviating oxidative stress and relieving inflammation. Reactive oxygen species (ROS) in cardiac tissues **(A)**. Superoxide dismutase (SOD) **(B)**, gluthathione (GSH) **(C)**, and malondialdehyde (MDA) **(D)** contents in cardiac tissues. The regulatory effect of Cana on cardiac mitochondria in myocardial tissues **(E)**. The scale bar is 5 and 1 μm in the upper panel and bottom panel, respectively. Serum concentrations of tumor necrosis factor (TNF)-alpha **(F)**, monocyte chemotactic protein 1 (MCP-1) **(G)**, and interleukin 6 (IL-6) **(H)**. The immunofluorescence staining of heme oxygenase 1 (HO-1) in cardiac tissues, scale bar: 50 μm **(I)**. Data are expressed as mean ± SEM. #*p* < 0.05, compared with the model group; *p < 0.05, compared with the control group. Six mice in each group were randomly selected for analyses.

Oxidative stress was increased in T2DM, and this appeared to underlie the development of T2DM and diabetic complications such as CVD (Tangvarasittichai, 2015). Oxidative stress is known as a major contributor to endothelial dysfunction (Magenta et al., 2013), which is a key precursor to the development of CVD (Rocha et al., 2010). The area and intensity of fluorescence from DHE oxidation were notably increased in the model group (*p* < 0.05) (Figure 2A), while Cana or Metf treatments markedly reversed this situation (*p* < 0.05). Notable decreases in the activities of SOD and GSH, while increasing MDA content were observed in the hearts of T2DM mice compared with the normal mice (Figures 2B–D). However, the above situation was conspicuously reversed by Cana supplementation, implying that Cana can effectively relieve myocardial oxidative stress (Figures 2B–D). Importantly, TEM revealed marked muscular fiber twisting, cardiomyocyte dissolution, intercalated disc blurred, and Z line disappearance. Meanwhile, some myofilaments were flocky in appearance and showed no cross striations in the myocardial tissues of diabetic mice. In addition, mitochondrial crista deformation and damage, mitochondrial autophagy (red arrows presented in Figure 2E, vacuoles with black residue), and fat drop (yellow arrows presented in Figure 2E, vacuoles without black spots) were remarkedly presented in the model group, the effects of which were greatly attenuated by Cana or Metf treatment (Figure 2E), which are beneficial for the suppression of mitochondrial ROS production and subsequently oxidative stress (Zhou et al., 2018).

Chronic inflammation is a mediator of vascular dysfunction in T2DM patients (de Jager et al., 2006; Du et al., 2015; Zhang et al., 2017). Through inflammatory processes, the initial lesion of atherosclerosis and CVD is formed (Libby, 2006). We found that HFD feeding elevated inflammation state by increasing serum levels of TNF-alpha, MCP-1, and IL-6 compared with the control group, whereas Cana administration markedly suppressed this increase in proinflammatory cytokines (Figures 2F–H). Besides, HO-1 level was remarkedly decreased in the model group, while Cana or Metf treatment effectively elevated the HO-1 level in T2DM mice (Figure 2I). Upregulation of HO-1 expression plays an important role in the protective response against oxidative injury and inflammatory effects (Lee and Chau, 2002), which proposes that HO-1 is a promising target protein for therapeutic intervention in CVD. In general, the above results indicate that Cana can effectively alleviate oxidative stress and systematic inflammation by protecting myocardial structural and mitochondrial homeostasis.

### Effect of Cana on hematological parameters in type 2 diabetes mellitus mice

Studies demonstrate the strong association between hematological parameters and risk of CVD during systemic inflammation, and hypoxemia is implicated in the pathophysiology mechanisms of CVD (Fan et al., 2018). Our results (Table 1) showed that the white blood cell (WBC) count, platelets, and mean platelet volume (MPV) levels were conspicuously increased in T2DM mice compared with the normal mice, while Cana or Metf treatment effectively reversed this adverse situation. There is a significant association of increased MPV and platelet counts with diabetes related to endothelial dysfunction, coronary artery disease, and its vascular complications (Jabeen et al., 2013; Lippi et al., 2015). Moreover, elevated WBC is a classical inflammatory marker and associated with several CVD risk factors (Yayla et al., 2017). The above results indicate that Cana can conduct an anti-CVD effect via regulating the disorderly hematological parameters.

**TABLE 1:** Effect of the canagliflozin (Cana) treatment on hematological parameters.

Variable	Control	Model	Metf	Cana
White blood cells (WBCs) (×10^9^/L)	4.6 ± 0.5	14.1 ± 0.2^*^	8.1 ± 0.9^*#^	10.0 ± 1.1^*#^
Monocytes (×10^9^/L)	0.2 ± 0.03	0.3 ± 0.02^*^	0.6 ± 0.04^*#^	0.4 ± 0.02^*#&^
Monocytes (%)	5.0 ± 0.3	3.8 ± 0.25^*^	7.4 ± 0.5^*#^	2.5 ± 0.3^*#&^
Lymphocytes (×10^9^/L)	3.2 ± 0.24	11.9 ± 0.9^*^	4.9 ± 0.35^*#^	11.2 ± 1.3^*&^
Lymphocytes (%)	67.4 ± 5.6	76.6 ± 4.3	60.8 ± 5.7^#^	83.7 ± 7.6^*#&^
Granulocytes (×10^9^/L)	1.3 ± 0.09	2.3 ± 0.14^*^	2.6 ± 0.15^*^	3.5 ± 0.41^*#&^
Granulocytes (%)	27.5 ± 2.7	19.5 ± 1.5^*^	31.8 ± 2.9^#^	13.7 ± 1.1^*#&^
Platelets (×10^9^/L)	1940.5 ± 124.2	3,307 ± 32.3^*^	2,467 ± 250.4^*#^	2,707 ± 265.1^*#^
PDW	17.4 ± 1.2	17.4 ± 1.3	18.0 ± 1.5	19.3 ± 1.2
MCV (fl)	49.5 ± 5.0	48.5 ± 5.0	48.7 ± 3.6	52.7 ± 4.8
RDW (%)	37.8 ± 3.2	38.5 ± 2.8	38.4 ± 3.4	37.0 ± 2.8
Mean platelet volume (MPV) (fl)	4.75 ± 0.5	7.56 ± 0.8^*^	6.0 ± 0.7^*#^	6.3 ± 0.7^*#^

Note. PDW, platelet distribution width; RDW, red blood cell distribution width; MCV, mean corpuscular volume. Data are expressed as mean ± SEM. ^&^ *p* < 0.05, the Cana group compared with the Metf group; #*p* < 0.05, compared with the model group; *p < 0.05, compared with the control group. Six mice in each group were randomly selected, and statistical analyses were performed.

### Effects of Cana on cardiovascular abnormalities in type 2 diabetes mellitus mice

**FIGURE 3:**
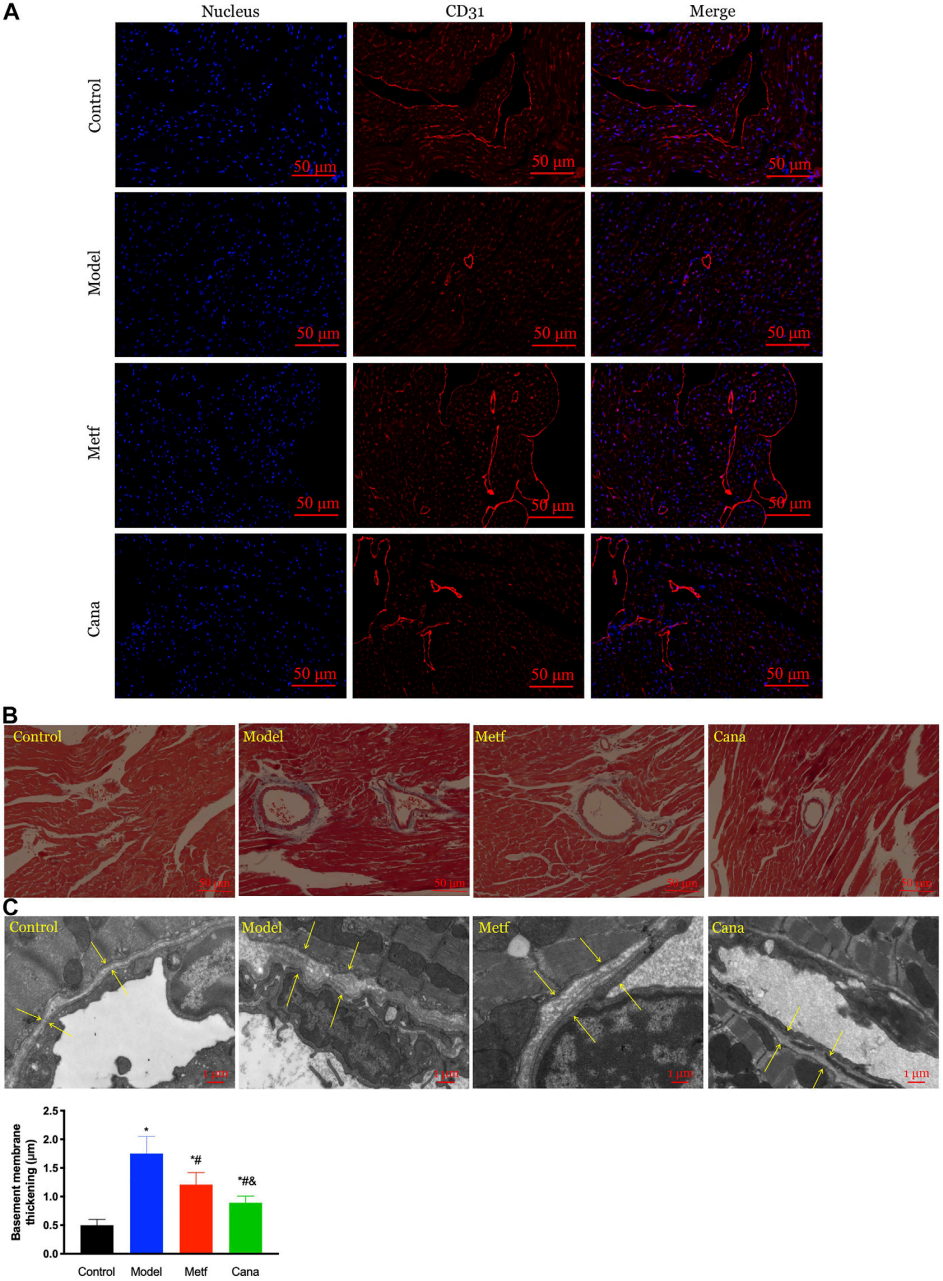
Effect of Cana on cardiovascular abnormalities in T2DM mice. The expression of cluster of differentiation 31 (CD31) in cardiac tissues **(A)**, immunofluorescence staining for CD31 (red), and nucleus (blue), scale bar: 50 μm. Masson’s trichrome staining in cardiac tissues **(B)**, scale bar: 50 μm. Changes in vascular basement membrane observed by transmission electron microscope (TEM) **(C)**, scale bar: 1 μm. ^&^ *p* < 0.05, the Cana group compared with the Metf group; ^#^ *p* < 0.05, compared with the model group; *p < 0.05, compared with the control group. Six mice in each group were randomly selected for analyses.

Normal vasculature is crucial to cardiac function and CVD, which is susceptible to hyperglycemia (Pinter et al., 1999). CD31 plays an important role in endothelial protection, which alleviates the apoptosis of vascular endothelial cells (Evans et al., 2001). HFD-induced T2DM triggered an overt drop in the number of CD31-positive microvessels compared with the normal mice (Figure 3A), while the unfavorable state was reversed by Cana or Metf treatment so as to play a good role in protecting the vascular endothelium. In addition, T2DM-induced basement membrane thickening and microvascular morphology including fibrosis (Figures 3B, C) were largely ameliorated by Cana administration. These data demonstrated a cardioprotective property of Cana by maintaining cardiovascular structural homeostasis.

### Effects of Cana on myocardial injury

**FIGURE 4:**
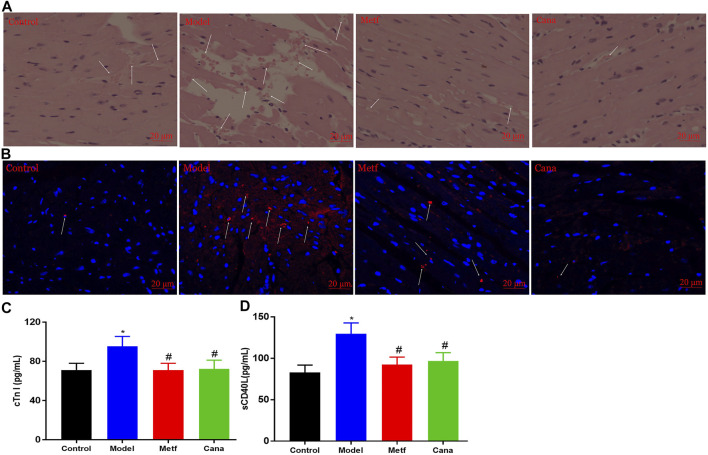
Effects of Cana on improving myocardial injury in T2DM mice. H&E staining in cardiac tissues **(A)**. TdT-mediated fluorescein nucleotide (cyanine 3-dUTP) nick-end labeling (TUNEL) assay to detect apoptosis in cardiac tissues **(B)**, blue represents nucleus, while red represents fragmented DNA, scale bar: 20 μm. Levels of cardiac troponin 1 (cTn I) **(C)** and soluble cluster of differentiation 40 ligand (sCD40L) **(D)** in serum. ^&^ *p* < 0.05, the Cana group compared with the Metf group; ^#^ *p* < 0.05, compared with the model group; *p < 0.05, compared with the control group. Six mice in each group were randomly selected for analyses.

Damage to microvessel integrity and cardiac microvascular endothelial cell are considered to be the initial step in vascular complications (Thygesen et al., 2018). Diabetes-induced effects (such as turbulent blood flow and capillary blockage) raised the accumulation of erythrocytes in the microvessel (Zhou et al., 2018), while Cana considerably retarded this negative situation (white arrows presented in Figure 4A). Additionally, Metf or Cana treatment inhibited the diabetes-induced TUNEL-positive cells from increasing (white arrows presented in Figure 4B), supporting the prosurvival capacity of Cana on hyperglycemia-mediated cardiac microvascular endothelial cell apoptosis. The above results indicated the beneficial effects of Cana on microvessel integrity, favoring a marked reduction in the risk of cardiac microvascular endothelial cell dysfunction or death in diabetic hearts.

Cardiac troponin is used to diagnose myocardial infarction, and the increased concentration of cardiac troponin indicates the elevated risk for adverse outcomes in individuals (Eggers and Lindahl, 2017). In addition, cTn I, an important subunit of cardiac troponin, is recognized as a marker of myocardial damage (Eggers and Lindahl, 2017). Studies also demonstrated that high levels of sCD40L indicate enhanced inflammatory responses, heightened risk of death, and myocardial infarction in patients (Schönbeck et al., 2001; Heeschen et al., 2003; Cipollone et al., 2003). In our study, levels of serous cTn I and sCD40L in T2DM mice were markedly elevated compared with the control group (Figures 4C, D), while Cana treatment markedly improved the situation. The above results demonstrate the salient effects of Cana on alleviating myocardial injury in T2DM mice.

### Cana modulated gut microbiota at various levels

**FIGURE 5:**
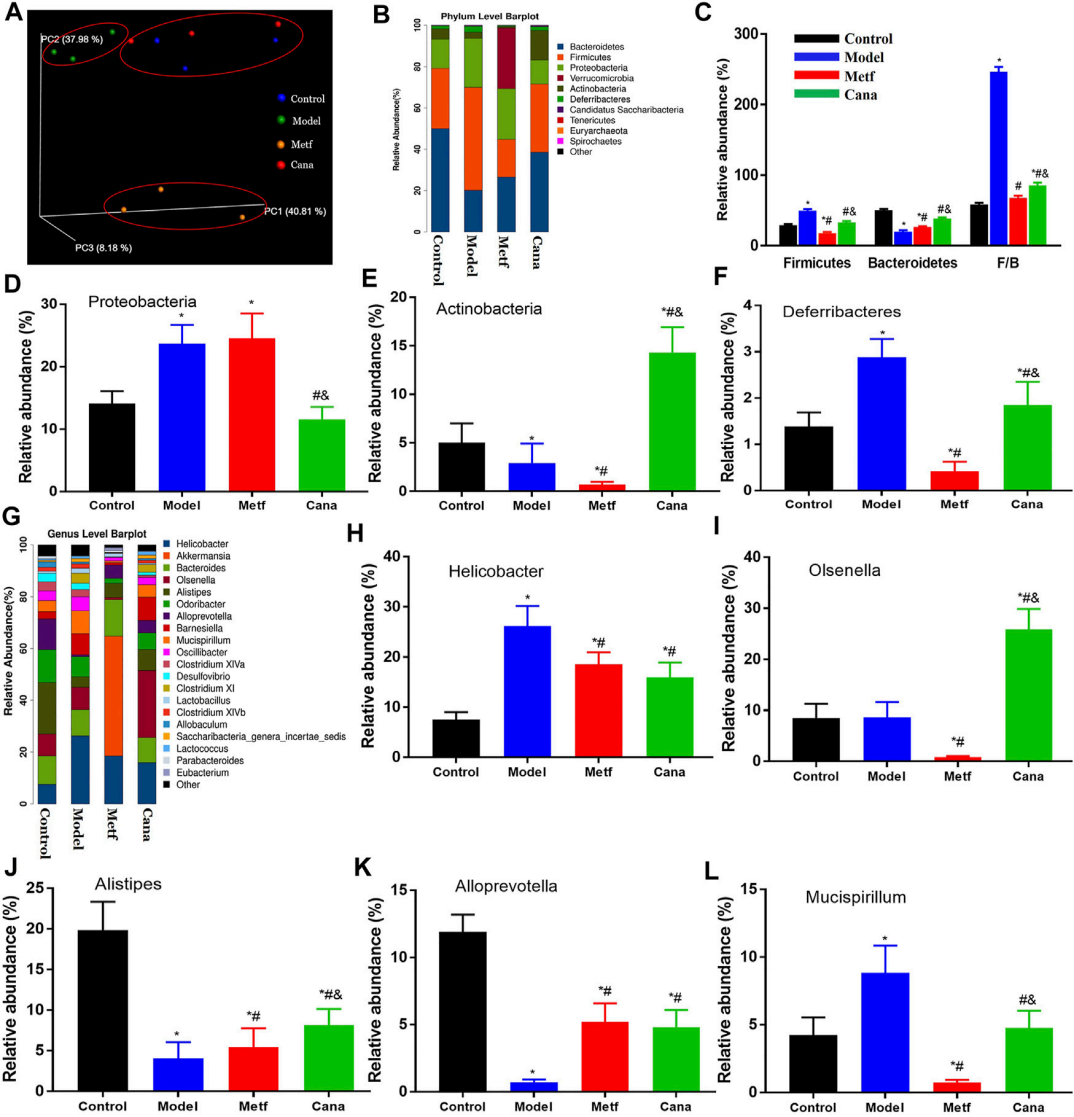
Modulation of microbiota by Cana treatment. PCoA plot of microbiota **(A)**. Abundance at the phylum level **(B)**, including Bacteroidetes and Firmicutes **(C)**, Proteobacteria **(D)**, Actinobacteria **(E)**, and *Deferribacteres* **(F)** in various groups. Abundance at the genus level **(G)**, including *Helicobacter* **(H)**, *Olsenella* **(I)**, *Alistipes* **(J)**, *Alloprevotella* **(K)**, and *Mucispirillum* **(L)**. Data are expressed as mean ± SEM. ^&^ *p* < 0.05, the Cana group compared with the Metf group; ^#^ *p* < 0.05, compared with the model group; *p < 0.05, compared with the control group. Three mice in each group were randomly selected for analyses.

As shown in Supplementary Table S1, α-diversity (e.g., Good’s coverage, Chao1, Shannon, and Simpson indices) of the colonic flora in the Cana-treated group was not markedly different compared with the control and model groups. In addition, evaluation of β-diversity based on nMDS ordination plot (Supplementary Figure S5A), weighted UniFrac heatmap (Supplementary Figure S5B), and weighted PCoA analysis (Figure 5A) showed that there were significant differences in colonic flora between the Cana treatment group and model group, and microbial community of the Cana group was similar to the control group. At the phylum level (Figure 5B), the Cana group had a higher abundance of Bacteroidetes with a lower abundance of Firmicutes, and an increased ratio of Firmicutes/Bacteroidetes (Figure 5C) compared with that of the model group. A significant elevation in the relative abundance of Proteobacteria was induced in the model group compared with control group and was remarkedly decreased in the Cana group, however, not reversed by Metf treatment (Figure 5D). In addition, administration of Cana remarkably enriched the abundance of Actinobacteria and decreased the abundance of Deferribacteres in mice with T2DM (Figures 5E, F). The abundance analysis at the genus level indicated that there were conspicuous differences in flora types and abundance in T2DM mice after Cana treatment (Figure 5G). Furthermore, Cana regulated the disorder of microbial community in the genus level as follows: 1. increasing the relative abundance of *Olsenella*, *Alistipes*, and *Alloprevotella* (Figures 5I–K), which may be associated with a healthier microbiome, as suggested previously (Kong et al., 2019); 2. decreasing the relative abundance of *Helicobacter* and *Mucispirillum* (Figures 5H, L), which was positively correlated with TNF-alpha and LPS contents (Li et al., 2019). Altogether, the above results demonstrated that intestinal ecosystem in mice with diabetic CVD were adjusted to a relatively normal state by oral administration of Cana.

### Cana induced phylotype changes in gut microbiota

**FIGURE 6:**
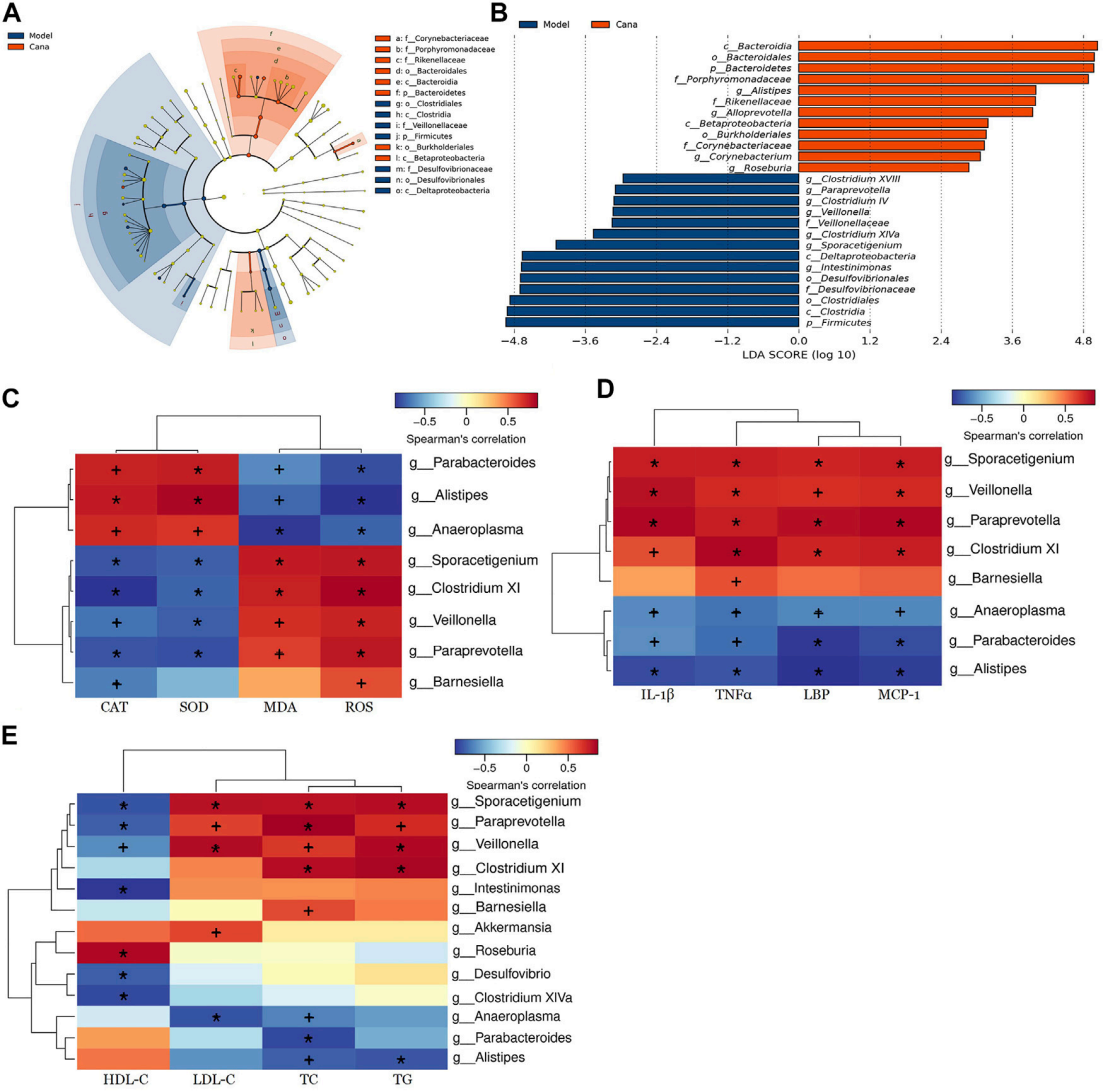
LEfSe and Spearman’s correlation analyses. LEfSe analyzed the most differential taxons between the model group and Cana treatment group **(A)**. Taxa increased after Cana treatment (orange) showed a positive score, while model group-enriched taxa (dark blue) showed a negative LDA score **(B)**. Heatmap of Spearman’s correlations analysis between bacteria and physiological indices **(C–E)**. The abscissa represents the physiological indices, and the ordinate represents the bacterial species. The shade of color directly indicates the correlation between physiological indices and bacteria. The correlation between biomarker and microbial species was visualized by color depth: the darker the color, the more relevant it is. *p < 0.01, +*p* < 0.05. Three mice in each group were randomly selected for analyses.

To further elucidate the effects of Cana treatment on the regulation of various bacterial taxa in the colon, a pairwise comparison between the model and Cana groups was conducted by LEfSe analysis. It indicated that Cana reduced the level of Firmicutes, including *Sporacetigenium*, *Veillonella*, and *Clostridium* XI, while remarkedly elevating Bacteroidetes, including short-chain fatty acid (SCFA) producers such as *Alloprevotella* and *Bacteroides* (*p* < 0.05) (Figures 6A,B). The Cana treatment increased the amount of Porphyromonadaceae, Rikenellaceae, and Corynebacteriaceae, while a decrease in Desulfovibrionaceae and Veillonellaceae. At the genus level, the Cana group was marked by a dramatic increase in *Alistipes*, *Roseburia*, and *Corynebacterium* (Figure 6B), which can increase the production of short-chain fatty acids (SCFAs) (Franzosa et al., 2018; Amabebe and Anumba, 2020). Meanwhile, *Paraprevotella*, *Veillonella*, *Sporacetigenium*, and *Intestinimonas* were significantly reduced after Cana treatment compared with the model group (Figure 6B). In general, the taxonomical distribution within groups at phylum, family, and genus levels for the colonic samples revealed the divergent composition of communities after Cana treatment in mice with diabetic CVD.

Using Spearman’s correlation analysis, relationships of physiological index and bacterial abundance were clarified (Figures 6C–E). At the genus level, *Parabacteroides* and *Alistipes* were positively correlated with SOD and CAT, while negatively correlated with IL-1β, MDA, ROS, TNF-alpha, MCP-1, and LBP levels, and their abundances were increased after Cana treatment. In addition, *Clostridium* XI, *Veillonella*, and *Paraprevotella* were negatively correlated with CAT and SOD, while positively related to MDA, ROS, and the above inflammatory cytokines levels. Meanwhile, *Alistipes* and *Roseburia* were negatively correlated with LDL-C, TC, and TG contents, while positively related to HDL-C level. Cana treatment caused an increase in the relative abundances of *Alistipes* and *Roseburia* in mice with diabetic CVD. Moreover, *Paraprevotella*, *Veillonella*, *Clostridium* XI, and *Barnesiella* presented a positive correlation with LDL-C, TC, and TG contents, while negatively related to HDL-C level. These findings indicate that alterations in the gut microbiota after Cana treatment lead to the reduction of systemic inflammation, oxidative stress, and lipid accumulation in mice with diabetic CVD. Collectively, these data reinforce the link between gut dysbiosis and diabetic CVD, and indicate that Cana can manipulate gut microbiota and attenuate the CVD complications of T2DM.

## Discussion

A clinical research indicated that Cana can lower the risk of cardiovascular events in T2DM patients (Perkovic et al., 2019). Meanwhile, the underlying mechanisms were still unclear. Our present study showed that Cana can markedly relieve the oxidative stress and inflammation state, which may be due to the Cana-maintained mitochondrial homeostasis of cardiac tissue in mice with diabetic CVD. Mitochondrial dysfunction resulted in the overproduction of ROS, and cardiovascular function can be improved by relieving mitochondrial dysfunction and its related oxidative stress and systemic inflammatory state (Ren et al., 2018). Further understanding of oxidative stress as well as its downstream effects on cellular function will be conducive to identify more specific targets for CVD treatment (Kayama et al., 2015).

Interestingly, Cana treatment decreased the relative abundance of Firmicutes, while remarkedly raising Bacteroidetes, such as *Alloprevotella* and *Bacteroides*, which were beneficial in alleviating hypertension and atherosclerosis (Tang et al., 2017). In addition, Cana administration was marked by a dramatic increase in *Roseburia*, *Alloprevotella*, and *Bacteroides*, which can elevate the production of SCFAs. More importantly, it has been recently confirmed that *Alistipes* was causally linked to decreased triglyceride concentration in serum (Liu et al., 2022), which was consistent with our present study (Figures 5J, 6E). Thus, gut microbiota may play a vital role in regulating serum lipid composition. Furthermore, Spearman’s correlation analysis demonstrated that the colonic microbiota composition altered by Cana treatment was related with the blood lipid, oxidative stress, and inflammation. It has been verified that gut microbiota and its metabolites affect the autophagy and damage of the mitochondria, thus, regulating the mitochondrial function of the host (Franco-Obregón and Gilbert, 2017). Therefore, Cana altered colonic microbiota composition compared with the model group in mice with T2DM-related CVD, which contributed to the improvement of mitochondrial homeostasis and the progress of CVD (Jin et al., 2021). It is worth noting that our present results indicated that the effects of Cana on alleviating diabetic CVD were mostly coincidental with that of the Metf group besides the effect on body weight lowering. It showed that Metf treatment significantly reduced the body weight compared with that of the Cana group (Supplementary Figure S3), which may relate with the various colonic microbiota regulation. Importantly, Metf markedly increased the abundance of *Akkermansia* compared with the Cana group (Figure 5G), which has been verified to decrease body weight in clinical trials (Shin et al., 2014; Depommier et al., 2019). Moreover, the bioavailabilities of Metf, Cana, Empa, and Dapa in humans were about 95%, 65%, 75%, and 78%, respectively (Beckmann, 1969; Garcia-Ropero et al., 2018). In addition, 81.2% and 15.3% of the oral intake of Empa were excreted with feces and urine in male mice as shown in the documents submitted to the FDA for approval (Application Number: 204629Orig1s000). Furthermore, 85.4% of Cana and its metabolites in male mice were excreted with the feces (Mamidi et al., 2014). While, Metf was mainly excreted through the urine and a small part through the feces (Beckmann, 1969). In general, the different ADME of the above drugs may result in various bioactivities.

**FIGURE 7:**
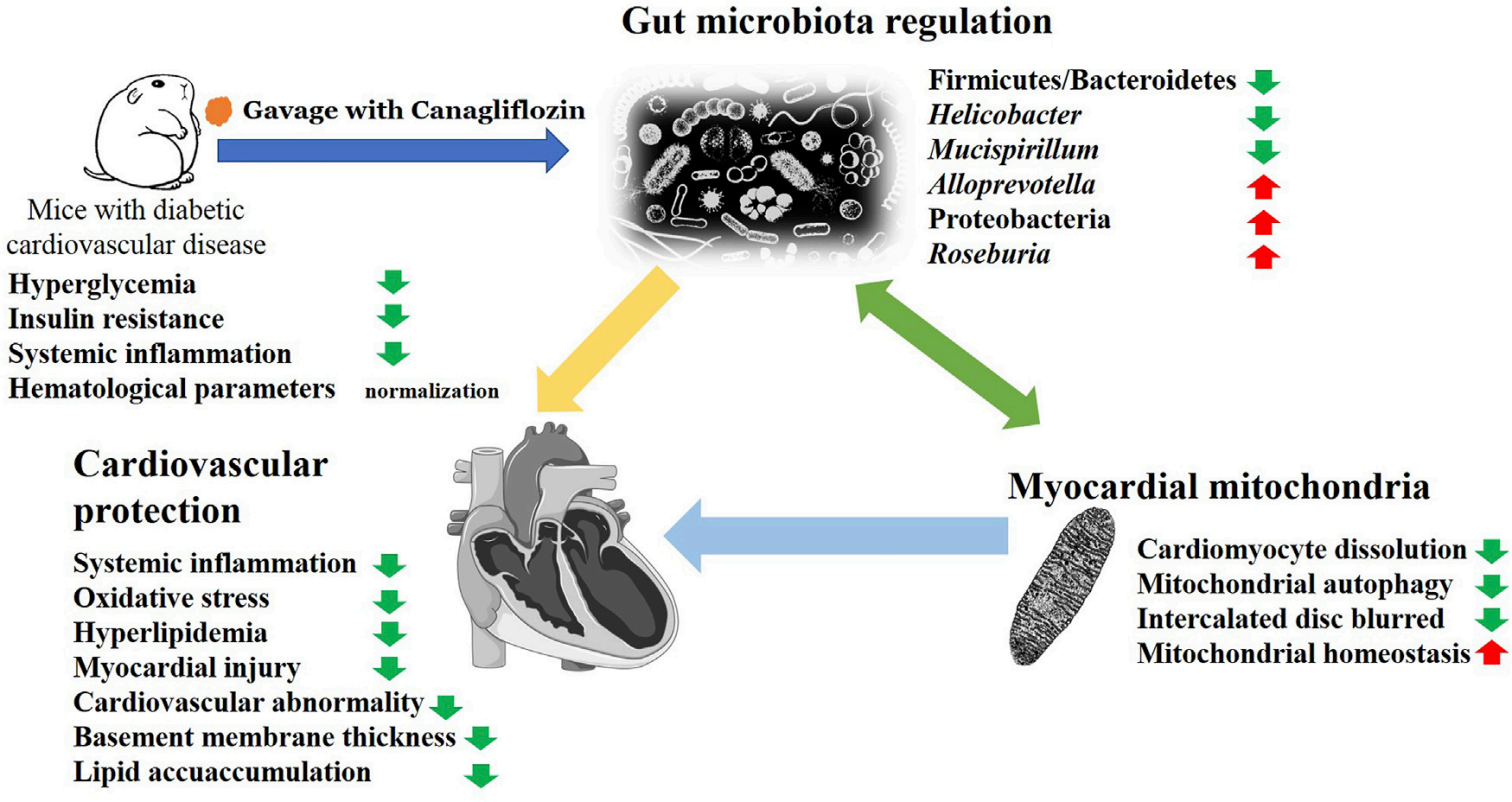
The possible mechanism of Cana on alleviating diabetic cardiovascular disease (CVD). In summary, Cana can effectively alleviate the risk factors for diabetic CVD such as oxidative stress, inflammation, hyperlipemia, atherosclerosis, mitochondrial disorder, thickness of vascular basement membrane, and myocardial injury. The amelioration of gut microbiota and myocardial mitochondrial homeostasis represent an important mechanism underlying the cardiovascular benefits.

In summary, the primary effects of Cana are its regulation on gut microbiota and myocardial mitochondrial homeostasis, which may play a considerable role in antiCVD (Figure 7). Altogether, our study presents a novel therapeutic role for Cana in T2DM-related CVD. Limitations to this study should be noted. First, further research to clarify the molecular mechanism of the associated changes in colonic microbiota that leads to the improvement of diabetic CVD is still necessary. Second, the relationship between microbiota and CVD should be further clarified utilizing fecal bacteria transplantation in antibiotic-treated or germ-free mice model, which is necessary to be comprehensively addressed in future research. In addition, the most important probiotic strains in alleviating diabetic CVD after Cana treatment were not clarified.

## Data Availability

The original contributions presented in the study are publicly available. The data can be found here: https://www.ncbi.nlm.nih.gov/sra/PRJNA795843.

## Glossary

T2DM: type 2 diabetes mellitus
CVD: cardiovascular disease
IR: insulin resistance
Metf: metformin
SGLT2i: sodium glucose cotransporter 2 inhibitors
Cana: canagliflozin
IPITT: intraperitoneal insulin tolerance test
ROS: reactive oxygen species;
OGTT: oral glucose tolerance test
TC: total cholesterol
TG: triglycerides
HDL-C: high-density lipoprotein cholesterol
LDL-C: low-density lipoprotein cholesterol
PDW: platelet distribution width
SOD: superoxide dismutase
MPV: mean platelet volume
AI: atherogenic index
AIP: atherogenic index of plasma
WBC: white blood cell count
FBG: fasting blood glucose
CD31: cluster of differentiation 31
MCV: mean corpuscular volume
HbA1c: hemoglobin A1c
RDW: red cell distribution width
GSH: glutathione
CAT: catalase
MDA: malondialdehyde
HOMA-IR: homeostasis model assessment-insulin resistance
MCP-1: monocyte chemotactic protein 1
IL-6: interleukin-6
TNF-alpha: tumor necrosis factor alpha
cTn I: cardiac troponin I
sCD40L: soluble cluster of differentiation 40 ligand
AMPK: AMP-activated protein kinase
TEM: transmission electron microscope
LefSe: linear discriminate analysis effect size
SEM: standard error of the mean
DHE: dye dihydroethidium
SCFAs: short-chain fatty acids
Dapa: dapagliflozin
Empa: empagliflozin
