# Supplementary material for: Canagliflozin Prevents Lipid Accumulation, Mitochondrial Dysfunction, and Gut Microbiota Dysbiosis in Mice With Diabetic Cardiovascular Disease
Source: Front Pharmacol. 2022 Feb 23;13:839640. doi: 10.3389/fphar.2022.839640 (PMC8905428; doi:10.3389/fphar.2022.839640)
Supplement: Supplementary file 1 [file DataSheet1.docx]

Supplementary Material

# Supplementary Tables and Figures

**Table S1** Diversity of colonic microbiota in different groups.

| Groups | Good’s coverage | Chao1 | Shannon indices | Simpson indices |
| --- | --- | --- | --- | --- |
| Control | 0.997981 | 275 | 5.63 | 0.9979 |
| Model | 0.998111 | 248 | 5.12 | 0.9282 |
| Metf | 0.997925 | 220^*^ | 4.08^*^ | 0.8663 |
| Cana | 0.998314 | 230 | 5.06 | 0.9291 |

Control, NCD-fed mice; Model, HFD-fed mice; Metf, HFD mixed with 225 mg/kg/d of metformin; Cana: HFD mixed with 50 mg/kg/d Cana. * *p* < 0.05, compared with the Control group; # *p* < 0.05, compared with the Model group; & *p* < 0.05, the Cana group compared with the Metf group. Three mice of each group were randomly selected to analyze.

**
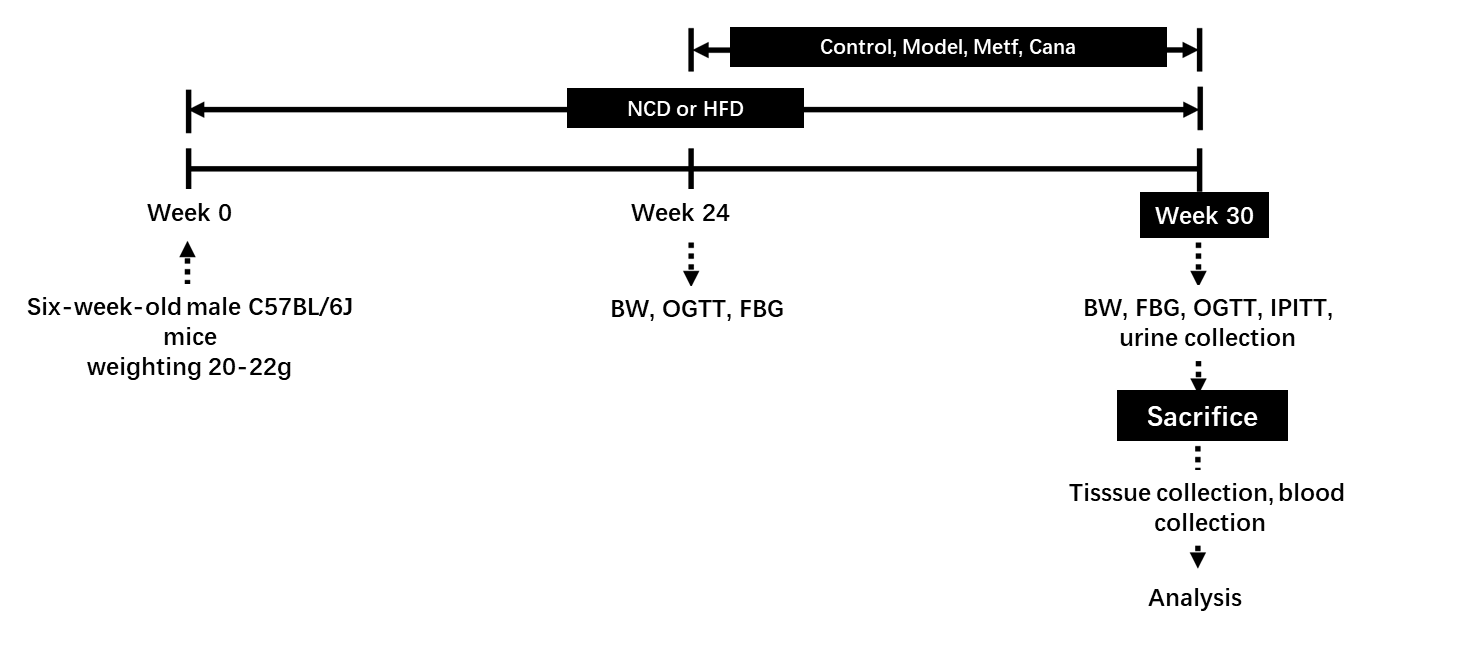
**

**Supplementary Figure 1.** **Schematic diagram of the expe****rimental protocol of this study**

Control, NCD-fed mice; Model, HFD-fed mice; Metf, HFD with 225 mg/kg/d metformin; Cana: HFD with 50 mg/kg/d Cana. BW: body weight; OGTT: oral glucose tolerance test; FBG: fasting blood glucose; IPITT: intraperitoneal insulin tolerance test.


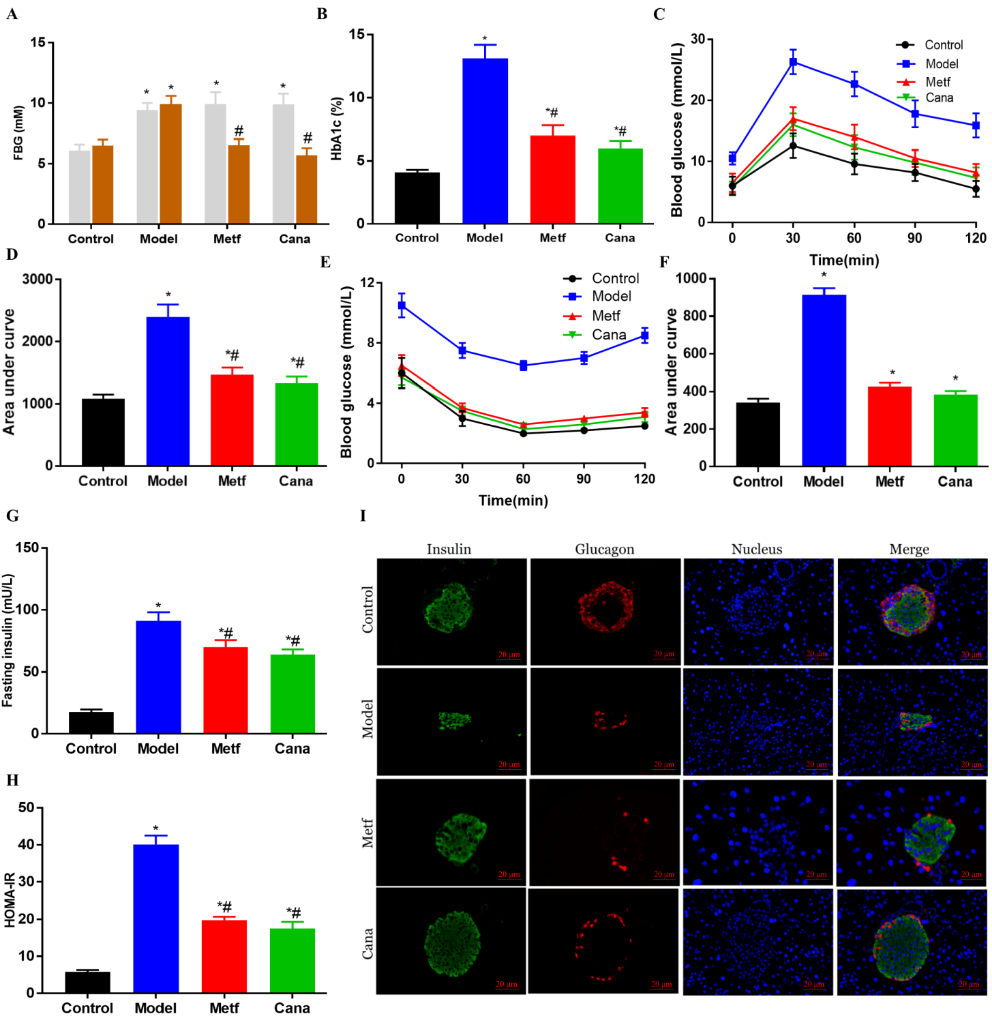


**Supplementary Figure 2. Cana treatment ameliorated glucose-insulin homeostasis**

FBG levels (A), grey and brown color represent the FBG at the 24^th^ week and at the 30^th^ week; HbA1c (B), Curve of OGTT (C), and areas under the curve (D) at the 30^th^ week; Curve of IPITT (E) and areas under the curve (F) at the 30^th^ week; Fasting insulin (G); HOMA-IR (H); Immunofluorescence staining for glucagon (red), insulin (green), and nucleus (blue) (I). Data are expressed as mean ± SEM. # *p* < 0.05, compared with the Model group; * *p* < 0.05, compared with the Control group. Six mice of each group were randomly selected to analyze.


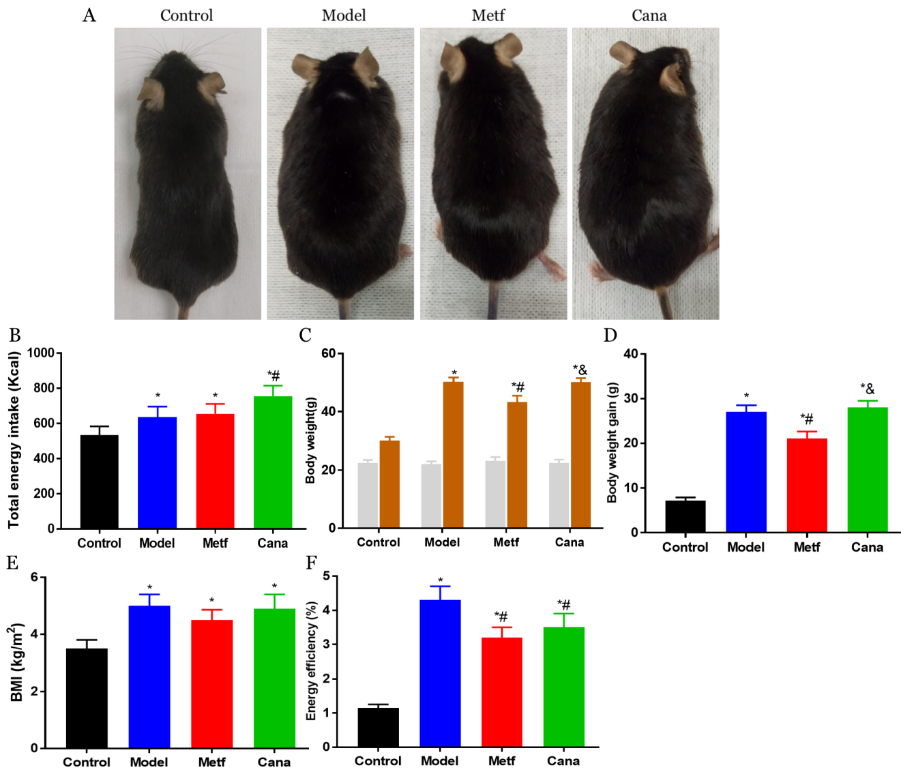


**Supplementary Figure 3. Effects of Cana treatment on physical parameters in T2DM mice**

The gross appearance feature of mice with different treatments (A); Total energy intake (B); Body weight (C), grey and brown color represent the body weight at the 24^th^ week and at the 30^th^ week; Body weight gain (D), BMI (E), and Energy efﬁciency (F) at the end of the trial. Data are expressed as mean ± SEM. & *p* < 0.05, the Cana group compared with the Metf group; # *p* < 0.05, compared with the Model group; * *p* < 0.05, compared with the Control group. Six mice of each group were randomly selected to analyze.


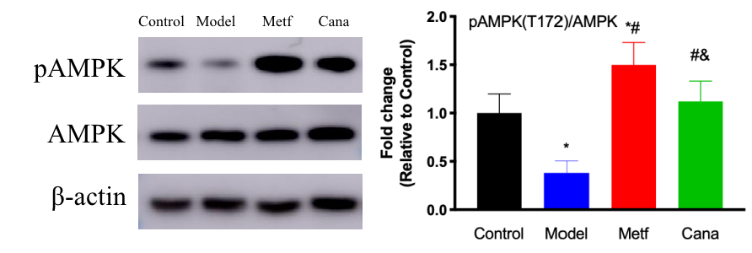


**Supplementary Figure 4. The activity of AMPK in cardiac tissues after various treatrments for 6 weeks.**

Control, NCD-fed mice; Model, HFD-fed mice; Metf, HFD with 225 mg/kg/d of metformin; Cana: HFD with 50 mg/kg/d Cana. Data are expressed as mean ± SEM. & *p* < 0.05, the Cana group compared with the Metf group; # *p* < 0.05, compared with the Model group; * *p* < 0.05, compared with the Control group. Three mice of each group were randomly selected, and statistical analyses were performed.


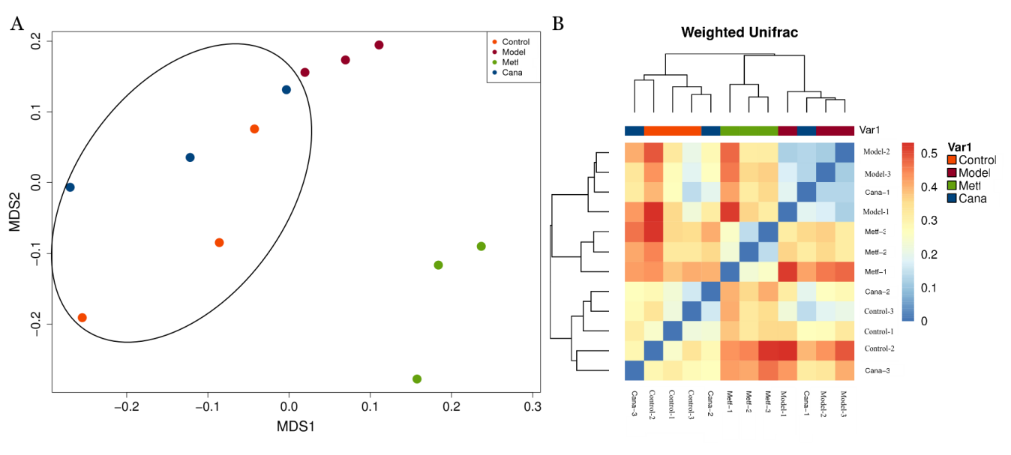


**Supplementary Figure 5. β-diversity of cecal microbiota among various groups**

Non-metric multidimensional scaling (nMDS) ordination plot (A) and weighted UniFrac heatmap (B). Control, NCD-fed mice; Model, HFD-fed mice; Metf, HFD with 225 mg/kg/d of metformin; Cana: HFD with 50 mg/kg/d Cana. Three mice of each group were randomly selected to analyze.
